# The effect of health facility delivery on neonatal mortality: systematic review and meta-analysis

**DOI:** 10.1186/1471-2393-13-18

**Published:** 2013-01-22

**Authors:** Gurmesa Tura, Mesganaw Fantahun, Alemayehu Worku

**Affiliations:** 1 Department of Reproductive Health and Health Service Management, School of Public Health, Addis Ababa University, Addis Ababa, Ethiopia; 2 Department of Epidemiology and Biostatistics, School of Public Health, Addis Ababa University, Addis Ababa, Ethiopia

**Keywords:** Neonatal mortality, Health facility delivery, Systematic review, Meta-analysis

## Abstract

**Background:**

Though promising progress has been made towards achieving the Millennium Development Goal four through substantial reduction in under-five mortality, the decline in neonatal mortality remains stagnant, mainly in the middle and low-income countries. As an option, health facility delivery is assumed to reduce this problem significantly. However, the existing evidences show contradicting conclusions about this fact, particularly in areas where enabling environments are constraint. Thus, this review was conducted with the aim of determining the pooled effect of health facility delivery on neonatal mortality.

**Methods:**

The reviewed studies were accessed through electronic web-based search strategy from PUBMED, Cochrane Library and Advanced Google Scholar by using combination key terms. The analysis was done by using STATA-11. I^2^ test statistic was used to assess heterogeneity. Funnel plot, Begg’s test and Egger’s test were used to check for publication bias. Pooled effect size was determined in the form of relative risk in the random-effects model using DerSimonian and Laird's estimator.

**Results:**

A total of 2,216 studies conducted on the review topic were identified. During screening, 37 studies found to be relevant for data abstraction. From these, only 19 studies fulfilled the preset criteria and included in the analysis. In 10 of the 19 studies included in the analysis, facility delivery had significant association with neonatal mortality; while in 9 studies the association was not significant. Based on the random effects model, the final pooled effect size in the form of relative risk was 0.71 (95% CI: 0.54, 0.87) for health facility delivery as compared to home delivery.

**Conclusion:**

Health facility delivery is found to reduce the risk of neonatal mortality by 29% in low and middle income countries. Expansion of health facilities, fulfilling the enabling environments and promoting their utilization during childbirth are essential in areas where home delivery is a common practice.

## Background

The fourth Millennium Development Goal (MDG_4_) calls for reducing the under-five mortality rate by two-thirds between 1990 and 2015. However, only four years reaming for the deadline, only 41% decline in under-5 mortality rate has been achieved globally till 2011. As a result, about 7 million children died before their 5^th^ birthday in the year 2011 worldwide. From these, about 5 million died before the age of one and nearly 3 million died within the first 28 days of birth. This indicated that 43% of under-five deaths and 60% of infant deaths were accounted by the neonatal mortality [1]. This pointed out that it is difficult to achieve the desired target for the reduction of infant and under-five mortality without particular focus on neonatal mortality.

More than 98% of these deaths occurred in the low and middle income countries. Sub-Saharan Africa is, by far, the region of the world with the highest level of child as well as neonatal morbidity and mortality and remained the most troubling geographic area. In this region, 1 in 9 children dies before age five, more than 16 times the average for the developed regions (1 in 152). Similarly, this region has the highest risk of death in the first month of life and is among the regions showing the least progress [1,2].

Most of these deaths were caused by infectious diseases, pregnancy-related complications, delivery-related complications including intra-partum asphyxia, birth trauma, and premature birth which can easily be prevented by skilled care during delivery and immediate neonatal period [1,3,4].

Skilled care during delivery has been recommended by World Health Organization (WHO) and the safe motherhood to improve the care provided to mothers during childbirth as it has a direct effect through prevention of infection, birth trauma, and asphyxia. Skilled care during labor is necessary to help normal things remain normal and to rapidly detect and deal with complications [5-8].

Similarly, many institutions and researchers have a doubt and have been questioning the safety of home delivery in the absence of skilled attendants. They strongly emphasize the importance of the place where a delivery takes place as complications that might arise during delivery need immediate action. They have a view that being at the right place at the time of delivery considerably increases the chances of neonatal survival [9,10].

On the other hand, currently, there is a shift to provide skilled delivery care at the community level including at home. In the developed countries’ setup such as, Australia, England and the Netherlands, it has been evidenced that safe delivery can be provided at home provided that the enabling environments are in place. As a result, many authors and institutions have been advocating that it is the right of women to choose where to give birth whether at home or at health facility [11-14].

However, in the middle and low-income countries’ setup where there are constraints of enabling environments, the quality and safety of cares are the areas of concern. To provide quality and safe delivery care, skilled attendants need supportive contexts in which to provide care. These include a supportive legal and regulatory framework, access to essential equipment and drugs, and a functioning referral system. As evidenced by safe motherhood studies in some low and middle income countries like Benin, Rwanda, Ecuador and Jamaica, the contribution of enabling factors and essential elements to health workers’ performance is critical [15-18]. Thus, thinking these enabling environments, the safety of home delivery is a great concern.

To come up with concrete evidence regarding the effect of health facility delivery on neonatal mortality, it is very important to have a systematic review and meta-analysis, particularly for the low and middle income countries. But, in recent years, even though there are some local studies, systematic review and meta-analysis of such studies are very scarce. The existing very few reviews were limited to the developed countries and compared only planned home births with planned hospital births. Moreover, they focused on perinatal outcomes and less attention to neonatal mortality [19,20].

Thus, the purpose of this systematic review and met-analysis was to determine the pooled effect of health facility delivery on neonatal mortality by reviewing a pool of evidences from studies conducted all over the world.

## Methods

### Search strategy and evaluation of studies

Studies for this review and meta-analysis were accessed through electronic web-based search by using EndNote software. To access the records the following combination key terms were used: place of birth AND neonatal mortality, place of delivery AND neonatal mortality, health facility delivery AND neonatal mortality and home delivery AND neonatal mortality. The main databases searched were PUBMED, Cochrane Library for systematic reviews and Advanced Google Scholar. WHO databases were also searched. After identifying key relevant articles their references were also looked into (ancestor search strategy). Similarly, other studies which cited them were looked on line (descendent search strategy).

### Inclusion criteria

•***Design*:** Because of ethical issues, Randomized Controlled Trial (RCT) Studies were limited on the review topic**.** As a result, all observational studies that assessed the relation between place of birth and neonatal mortality were included.

•***Publication status*:** Both published and unpublished or grey literatures including Master’s and other thesis were included.

•***Language*:** Only articles published and grey literatures reported in English language were included because of inability to read and understand other languages.

•***Publication or report year*:** Though 5-10 years back is preferred for systematic review and meta-analysis, publications or reports made from January 1980-October 2012 were identified here because of the limited number of existing studies on the topic that best fit for the review.

### Exclusion criteria

Articles in which the exposure and outcome variables were not clearly indicated were excluded. In addition, studies that did not use appropriate sample size determination or sampling methods and studies that compared planned hospital births (with high risk) and planned home births (low risk) or provided particular intervention for home delivery and used this intervention as a means of classification were also excluded.

### Data abstraction

This review was conducted from October 15-30, 2012. The review was conducted in accordance with the Preferred Reporting Items for Systematic Review and Meta-Analyses (PRISMA) statement having 27 items Checklist [21]. The relevance of the reviewed studies was checked based on their title, objectives, methods and key variables. Initially, by using the above stated combination key terms, studies conducted on topics related to the review title were retrieved.

Then, after excluding duplicated retrievals, studies or reports not found to be relevant for the review were excluded. For the rest, abstracts were accessed and screened based on the independent and dependent variables under review (place of birth and neonatal mortality). Studies that were found to be non-relevant were excluded during this screening. Full text articles or reports were accessed for the reaming. Based on the preset inclusion and exclusion criteria, eligibility of the studies was assessed. Two of the authors independently conducted the review and consensus was reached through discussion when there were differences. Some articles did not have adequate data in which case the corresponding authors were contacted and necessary data were obtained.

### Data analysis

The necessary information was extracted from each original study by using a format prepared in Microsoft Excel spreadsheet and transferred to STATA/SE for windows version 11 for the meta-analysis. Heterogeneity among the original studies was checked by using I^2^ test statistic. As the test statistic showed significant heterogeneity among studies (I^2^ = 97%, p<0.001) in the fixed-effects model, random-effects model was used to estimate the DerSimonian and Laird's pooled effect. The pooled effect was expressed in the form of relative risk.

Publication bias was checked by using funnel plot asymmetry and statistical significance test by Begg’s rank correlation and Egger’s linear correlation in random-effects model. As the results of the test suggested possible existence of significant publication bias (p=0.01 in Egger’s test), the final effect size was determined by applying trim and fill analysis in the random-effects model.

## Results

### Description of original studies

A total of 2,330 records related to the review topic were accessed. After removing duplicated retrievals, 2,216 records remained, of which 1,942 were excluded during the initial assessment as their titles were found to be non-relevant. For the remaining 274 records, abstracts were accessed and screened. However, 237 were excluded because, the abstracts were not relevant based on the exposure and outcome variables. As a result, 37 full text articles/reports were accessed and assessed for eligibility based on the pre-set criteria. Finally, 19 studies fulfilled the eligibility criteria and included in the qualitative systematic review and quantitative meta-analysis.

Six studies, a case control study in Iran [22], secondary data analysis from Demographic and Health Survey (DHS) in Pakistan [23], RCT in Nepal [24], secondary data analysis in Bangladesh [25], case control study in Zimbabwe [26], and population based retrospective study in Haryana, India [27] were excluded because they did not have enough information for the meta-analysis, i.e., the number of total live births and number of neonatal deaths were not separately indicated and compared for facility delivery and home delivery.

Eight studies, prospective cohort studies in North America [28], Canada [29-31], England [32], Sweden [33], cross-sectional study in England and Wales [34] and secondary data analysis in the Netherlands [35] were excluded because these studies compared planned home births (assumed to have low risk) and planned facility deliveries (high risk) which were different from what this review and analysis intended to compare. This review intended to measure the difference in the occurrence of neonatal mortality regardless of certain interventions based on certain risks. In addition, these studies tried to measure perinatal mortality as primary outcome which is different from the outcome for this review, neonatal mortality.

**Figure 1:**
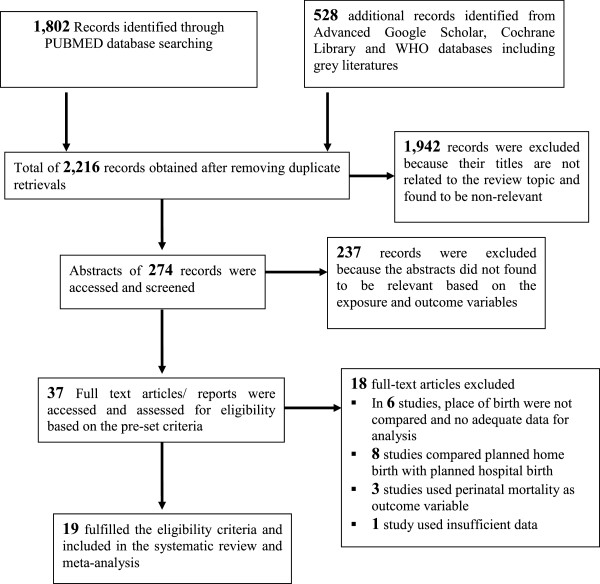
Flow chart showing the procedure of selecting studies for the systematic review and meta-analysis, 1988-2012.

Three studies, secondary data analysis in Democratic Republic of Congo [36], DHS analysis in Indonesia [37], and cross-sectional study in Bangladesh [38], were excluded because the outcome measure was perinatal mortality which is different from the outcome measure of this review (neonatal mortality). One cohort study in Bangladesh [39] was also excluded because of some methodological limitations. The study compared 917 home deliveries with 17 health facility deliveries. During the review, the authors hesitated for the sufficiency of the 17 health facility delivery for comparison with the 917 home deliveries and decided to exclude (Figure 1).

Out of the 19 studies that were eligible and included in the systematic review and meta-analysis, almost all (18/19) were from low and middle income countries (Africa and Asia). Nine were from Africa: Nigeria [40,41], Uganda [42], Malawi [43], Egypt [44], Ghana [45], Tanzania [46], Ethiopia [47] and Burkina Faso [48]. Nine were from Asia: China [49], India [50,51], Iran [52], Vietnam [53,54], Indonesia [55] and Pakistan [56,57]. Only one was from Europe, Italy [58]. The publication year of these studies ranged from 1988-2012. However, the majority (14/19) of the studies were published in the last five years (2008-2012).

Regarding study design, nine were cross-sectional, five were prospective cohort study, four were case-control study, and only one was community trial. The original sample size for each study ranged from 300 in case-control study in India to 898,360 in cross-sectional study in China.

In all the 19 studies included in the review and meta-analysis, a total of 1,606,805 live births were involved. Of whom, 18,186 died within 28 days of birth, making weighted neonatal mortality rate to be 11.32 per 1000 live births. When this is stratified by the place of birth, 1,504,450 were born in health facilities among whom, 14,821 died within 28 days of birth, making weighted neonatal mortality rate among facility deliveries to be 9.85 per 1000 live births. Whereas, 102,355 were born at home among which 3,365 died within 28 days of birth, making weighted neonatal mortality rate among home deliveries to be 32.88 per 1000 live births (Table 1).

**Table 1:** List of 19 studies included in the meta-analysis on the effect of health facility delivery on neonatal mortality, 1988-2012

**S/N**	**Author(s) & year of publication/report**	**Country**	**Design**	**Sample size**	**Health facility**		**Home**	
					**Live-births**	**Neonatal deaths**	**Live-births**	**Neonatal deaths**
1	Feng *et al*., 2011	China	Cross-sectional	898,360	840,622	6,592	57,738	1,664
2	Parazzine *et al*., 1988	Italy	Cross-sectional	638,438	622,381	6,488	16,057	275
3	Owa *et al*., 1998	Nigeria	Cross-sectional	7,225	5,741	653	1,484	285
4	Nathan *et al*., 2012	Tanzania	Prospective cohort	8,593	5,146	188	3,447	111
5	McDermott *et al*., 1996	Malawi	Prospective cohort	3,860	2,251	131	1,609	133
6	Okantey, 2008	Ghana	Cross-sectional	536	264	69	272	107
7	Titaley *et al*., 2008	Indonesia	Cross-sectional	15,800	5,948	96	9,852	152
8	Sharifzadeh *et al*., 2008	Iran	Case Control	468	227	68	241	88
9	Nga *et al*., 2012	Vietnam	Community trial	14,453	13,003	161	1,450	72
10	Upadhyay *et al*., 2012	India	Nested case control	5,444	2,871	102	2,573	84
11	Malqvist *et al*., 2010	Vietnam	Case control	782	599	80	183	58
12	Oti *et al*., 2011	Nigeria	Cross-sectional	5,708	2,009	65	3,699	122
13	Joshi, 2003	India	Case control	300	126	27	174	73
14	Tesfaye 2003	Ethiopia	Cross-sectional	1,462	837	27	625	41
15	Jehan *et al*., 2009	Pakistan	Prospective cohort	1,121	893	43	228	10
16	Dialo *et al*., 2011	Burkina Faso	Prospective cohort	864	308	10	556	30
17	Nankabirwa *et al*., 2011	Uganda	Prospective cohort	835	490	7	345	11
18	Ayzen *et al*., 2010	Pakistan	Cross-sectional	565	317	11	248	4
19	Seedhom *et al*., 2008	Egypt	Cross-sectional	1,991	417	3	1,574	45
			**Total**	**1,606,805**	**150,4450**	**14,821**	**102,355**	**3,365**

### Pooled effect size

**Figure 2:**
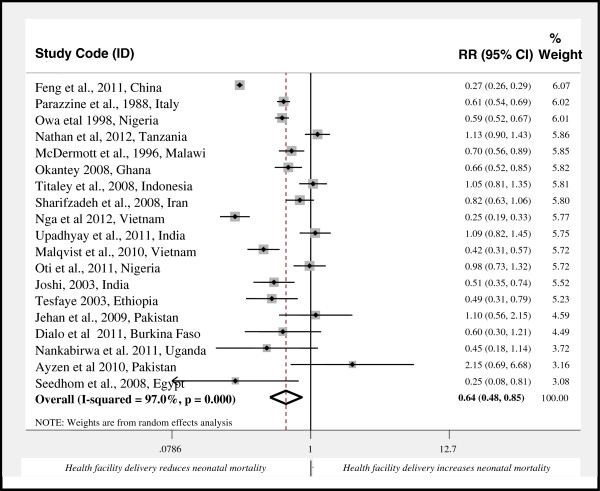
Forest Plot of 19 studies on the effect of health facility delivery on neonatal mortality, 1988-2012.

The pooled effect size of neonatal mortality among health facility delivery in the form of relative risk was 0.40 (95% CI: 0.39, 0.42) as compared to home delivery in the fixed effects model. However, the I^2^ test showed significant heterogeneity among studies (I^2^ = 97.0%, p < 0.001). As a result, random effects model was used to determine the effect size. In this model, among the 19 studies included in the analysis, 10 showed statistically significant association between place of delivery and neonatal mortality and the rest 9 showed non-significant. The pooled effect size by the random-effects model became 0.64 (95% CI: 0.48, 0.85) for health facility delivery as compared to home delivery (Figure 2).

**Figure 3:**
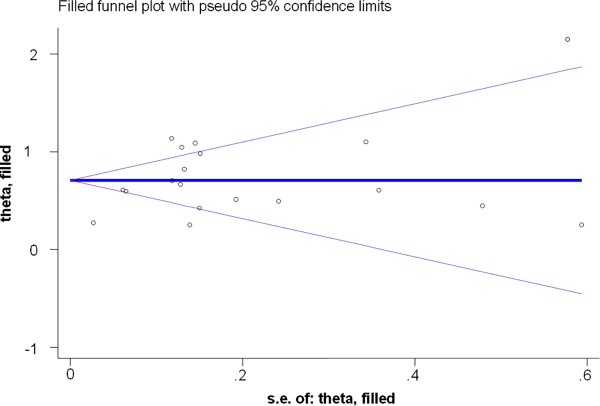
Filled funnel plot of the 19 studies included in the meta-analysis on the effect of health facility delivery on neonatal mortality, 1988-2012.

Publication bias was checked by using funnel plot asymmetry as well as Begg’s and Egger’s test of significance. On visual observation, the funnel plot found to be asymmetric. But, the Begg’s test showed no significant rank correlation with Kendall’s score of −31 and p=0.28. This did not support the funnel plot asymmetry, probably because of small number of studies. As a result, Egger’s test of linear correlation for absolute test was considered. This showed positive significant liner correlation with r = 2.81 (95% CI: 1.10, 4.52) and p=0.003, suggesting significant publication bias. This highlighted that studies with larger sample sizes having larger effect sizes might have been published and included in the review and meta-analysis. As a result, trim and fill analysis was done to adjust the final effect size. After trim and fill in the Random-effects model, the final pooled effect size was 0.71 (95% CI: 0.54, 0.87) with p<0.001. This shows that there is a significant difference in the rate of neonatal mortality between neonates born at health facility and at home (Figure 3).

To identify the possible causes of heterogeneity, stratified analysis was done based on the study designs, sample size and proportion of health facility delivery. In the fixed effects model, except cohort study, all the designs found to show significant effects. However, in the random effects model, cross-sectional studies and community trail studies were found to show significant effect whereas case-control studies and cohort studies did not show significant effects. The stratified analysis also revealed that there were differences in the effect size as the sample size differs. The analysis showed that the higher the sample size (>10,000) the stronger the effect size (Table 2).

**Table 2:** Stratified analysis of the 19 studies included in meta-analysis based on study designs, sample size and proportion of health facility deliver, 1988-2012

**Stratifying variable**	**Sample size**	**Fixed-effects RR (95% CI)**	**Random-effects RR (95% CI)**
**Study design**			
Prospective cohort	15,273	0.87 (0.75, 1.82)	0.82 (0.59, 1.14)
Case-Control	6,994	0.70 (0.60, 0.81)	0.67 (0.43, 1.03)
Cross-Sectional	1,570,085	0.37 (0.35, 0.38)	0.63 (0.41, 0.95)
Community trial	14,453	0.23 (0.19, 0.33)	0.23 (0.19, 0.33)
**Sample size**			
<1,000	4,350	0.62 (0.54, 0.71)	0.62 (0.47, 0.81)
1,000-5,000	8,434	0.63 (0.52, 0.78)	0.56 (0.36, 0.85)
5,001-10,000	26,970	0.76 (0.69, 0.83)	0.91 (0.62, 1.33)
>10,000	1,567,051	0.32 (0.31, 0.34)	0.46 (0.25, 0.83)
**% of health facility delivery**			
<50%	25,667	0.80 (0.71, 0.90)	0.74 (0.59, 0.94)
≥50%	1,581,138	0.37 (0.36, 0.39)	0.60 (0.43, 0.87)

Similarly, the difference in the coverage of health facility delivery resulted in variation in effect size. When proportion of health facility delivery is less than 50%, the effect size becomes 0.74 (95% CI: 0.59, 0.94), when the proportion of health facility delivery is 50% or above the effect size becomes 0.61 (95% CI: 0.43, 0.87) in the random effects model, however, in the fixed effects model this variation is much more significant (Table 2). With this, the difference in study designs, the difference in sample sizes and the difference in the proportion of health facility delivery are likely to be the causes for the heterogeneity. It was also planned to stratify based on level of development as high income countries and middle and low income countries. However, nearly all (18/19) were from the middle and low income countries and this could not be done.

## Discussions

This systematic review and meta-analysis tried to assess the pooled effect of health facility delivery on neonatal mortality.

The findings revealed that health facility delivery has statistically significant effect on neonatal mortality. It has resulted in 29% reduction in risk of neonatal mortality. As nearly all of the studies included in meta-analysis were from low and middle income countries, this figure can best applies for these countries. This effect had also been observed in some prior reviews. The systematic review and Delphi estimation conducted on more than 20 studies in developing countries showed that comprehensive emergency obstetric care and basic emergency obstetric care resulted in a reduction of intra-partum related neonatal deaths by 85% and 40% respectively [59].

Individual country’s experience also supports this finding. Portugal [60] and Chile [61] for example have shown significant reduction in neonatal mortality by expanding obstetric facilities and increasing the coverage of health facility delivery. This could be because of the fact that clean and safe delivery can be given at health facility. This in turn avoids trauma, infection and other risks that lead to morbidity and mortality of neonates.

Even though, a total of 2,216 studies were accessed, 37 original studies fit to the review topic and only 19 of them fulfilled the selection criteria. Among the 19 included studies, 14 were published in the last five years. This shows that the issue of neonatal mortality reduction through institutional delivery is a relatively recent research agenda. Because of the ethical issues, RCT studies were almost non-existent on this topic. As a result, observational studies were included. Many authors witnessed that observational studies can give valid findings with moderate effects when RCTs are not available to provide strong evidences [62-64]. With this, the finding of this systematic review and meta-analysis is taken as valid in showing moderate evidence.

The stratified analysis showed that the effect is higher in areas where the coverage of health facility delivery is high. When health facility delivery converge is above 50%, there is a reduction of about 40% in neonatal mortality as compared to 26% reduction when health facility delivery is less than 50%. This might be because, in areas where there is low coverage of health facility delivery, women usually give birth at home and go to the health facility after encountering some problems during labor. As a result, the child to be born is more likely to have some health problems and die during the neonatal period.

For program implication, in middle and low income countries, the issues of enabling environments need special emphasis while promoting home delivery. Because, in areas where there is shortage of equipments, drugs and other supplies together with problem of emergency referral, the safety of home delivery in reducing neonatal mortality may be under question. Thus, in such areas encouraging women to give birth in health facilities where necessary enabling environments are in place is very essential. Moreover, health facility delivery will also create an opportunity for the mother and the newborn to receive immunization and other necessary health information on preventive measures that may have an effect on preventing neonatal death.

Because of the variation in the design, sample size and the proportion of health facility delivery, significant heterogeneity among the studies was observed. As a result, random effects model was used to estimate the final pooled effect. Similarly, because of the existence of significant publication bias, trim and fill analysis was used. These might have underestimated the true effect of health facility delivery on neonatal mortality. So, it is important to note of this while interpreting and using this findings.

This systematic review and meta-analysis may have limitations as it was limited to publications and reports made in English language and observational studies. In addition, because of the nature of the meta-analysis that uses aggregated group data, the skill of the delivery attendant and other confounding factors were not controlled. This might have affected the effect size. Therefore, the findings of this systematic review and meta-analysis should be interpreted in the context of both inherent limitations of the original studies and the current reviews and analysis.

## Conclusions and recommendations

This meta-analysis found statistically significant association between place of delivery and neonatal mortality. In the low and middle income countries, health facility delivery was found to reduce the risk of neonatal mortality by 29%. Therefore, expansion of health facilities and promotion of their utilization are essential in areas where home delivery is a common practice and enabling environments are scarce. In addition, longitudinal studies need to be encouraged in areas where studies are lacking to come up with a more precise effect.

## Competing interests

The authors declare that they do not have any competing interest concerning the findings of the study.

## Authors’ contributions

TG involved in the conception of the study. TG, FM and WA conducted the review and screened the records for eligibility. TG carried out data extraction and conducted statistical analysis under the supervision of FM and WA. TG prepared the initial report which latter be read and edited by FM and WA. The final manuscript were read and approved by all authors.

## Pre-publication history

The pre-publication history for this paper can be accessed here:

http://www.biomedcentral.com/1471-2393/13/18/prepub
